# Increased therapeutic potential of an experimental anti-mitotic inhibitor SB715992 by genistein in PC-3 human prostate cancer cell line

**DOI:** 10.1186/1471-2407-6-22

**Published:** 2006-01-24

**Authors:** David A Davis, Sarah H Sarkar, Maha Hussain, Yiwei Li, Fazlul H Sarkar

**Affiliations:** 1 Departments of Pathology, Karmanos Cancer Institute, Wayne State University School of Medicine, Detroit, MI, USA; 2 Department of Natural Sciences, University of Michigan-Dearborn, Michigan, Ann Arbor, MI, USA; 3 Division of Hematology/Oncology, Department of Internal Medicine, University of Michigan, Ann Arbor, MI, USA

## Abstract

**Background:**

Kinesin spindle proteins (KSP) are motor proteins that play an essential role in mitotic spindle formation. *HsEg5*, a KSP, is responsible for the formation of the bipolar spindle, which is critical for proper cell division during mitosis. The function of *HsEg5 *provides a novel target for the manipulation of the cell cycle and the induction of apoptosis. SB715992, an experimental KSP inhibitor, has been shown to perturb bipolar spindle formation, thus making it an excellent candidate for anti-cancer agent. Our major objective was a) to investigate the cell growth inhibitory effects of SB715992 on PC-3 human prostate cancer cell line, b) to investigate whether the growth inhibitory effects of SB715992 could be enhanced when combined with genistein, a naturally occurring isoflavone and, c) to determine gene expression profile to establish molecular mechanism of action of SB715992.

**Methods:**

PC-3 cells were treated with varying concentration of SB715992, 30 μM of genistein, and SB715992 plus 30 μM of genistein. After treatments, PC-3 cells were assayed for cell proliferation, induction of apoptosis, and alteration in gene and protein expression using cell inhibition assay, apoptosis assay, microarray analysis, real-time RT-PCR, and Western Blot analysis.

**Results:**

SB715992 inhibited cell proliferation and induced apoptosis in PC-3 cells. SB715992 was found to regulate the expression of genes related to the control of cell proliferation, cell cycle, cell signaling pathways, and apoptosis. In addition, our results showed that combination treatment with SB715992 and genistein caused significantly greater cell growth inhibition and induction of apoptosis compared to the effects of either agent alone.

**Conclusion:**

Our results clearly show that SB715992 is a potent anti-tumor agent whose therapeutic effects could be enhanced by genistein. Hence, we believe that SB715992 could be a novel agent for the treatment of prostate cancer with greater success when combined with a non-toxic natural agent like genistein.

## Background

Prostate cancer is one of the leading causes of cancer fatality in the United States amongst males [1]. Development of chemotherapeutic agents to induce apoptosis of tumor cells with lower toxicity in patients is currently being investigated by many scientists. Many of these agents have been synthetically engineered or derived from natural plant products. Genistein, a predominant soy isoflavone, has been shown to inhibit proliferation in tumor cells *in vitro *and *in vivo *without any visible toxicity to normal cells [2,3]. In addition, individuals with diets high in soy show considerably lower incidences of prostate cancer [4]. At the molecular level, genistein is known as a protein tyrosine kinase inhibitor and has been shown to alter the expression of genes, which are critical for the control of cell proliferation, apoptosis, and cell signalling [5]. Tyrosine kinase is involved in many multi-cellular aspects of an organism [6]. In prostate epithelial cells, tyrosine kinase regulates cell-to-cell signaling that regulates growth, differentiation, adhesion, motility and programmed cell death, which ultimately play significant roles in the manifestation of human disease states such as diabetes and cancer [6]. With this information at hand, we studied the growth inhibitory effects of genistein with a new experimental anti-mitotic agent SB715992, chemically defined as *n-(3-amino-propyl)-n- [R-1-(3-benzyl-7-chloro-4-oxo-3, 4-dihydro-quinazolin-2-yl)-2-methyl-propyl]-4-methyl-benzanide methanesulfonate *(C_30_H_33_ClN_4_O_2_CH_4_O_3_S).

SB715992 is a kinesin spindle protein (KSP) inhibitor whose cellular effects may provide a novel treatment for cancer. Human KSP, encoded by *Hs*Eg5, has been shown to localize along interpolar spindle microtubules and at the spindle poles. KSP plays a rigid role in cell mitosis and is required for cell cycle progression. It mediates centrosome separation and formation of the bipolar mitotic spindle, which is important for cell mitosis. Inactivation of KSP caused improper cell division and cell cycle arrest during mitosis, ultimately leading to apoptotic cell death [7]. In this study, we investigated the cellular and molecular effects of SB715992 treatment alone and in combination with genistein on PC-3 human prostate cancer cells *in vitro*.

## Methods

### Cell culture and reagents

PC-3 human prostate cancer cells (ATCC, Manassas, VA, USA) were cultured in RPMI 1640 medium (Invitrogen, Carlsbad, CA, USA) with 10% fetal bovine serum in a 5% CO_2 _atmosphere at 37°C. Kinesin spindle protein inhibitor, SB715992, (GSK, GlaxoSmithKline, UK) was dissolved in distilled water to prepare a 1 μM stock solution. SB715992 was then applied directly to RPMI 1640 medium in experimental cultures at varying concentration. Tyrosine kinase inhibitor, genistein (Toronto Research Chemicals, North York, Ontario, Canada), was dissolved in sterile 0.1 M Na_2_CO_3 _to prepare a 10 mM stock solution. Subsequently, genistein was also applied directly to RPMI 1640 medium at the concentration of 30 μM in experimental cultures.

### Cell inhibition assay

PC-3 prostate cancer cells were seeded in 96 well plates at a density of 4 × 10^3 ^cells/well. PC-3 cells were incubated for 24 hours to allow attachment to the surface of each well of the tissue culture plate. Then, the cells were treated with varying concentration of reagents and incubated for 1 to 3 days. First, PC-3 cells were treated with 15 and 30 nM of SB7159992, respectively. Second, PC-3 cells were subjected to combinational treatments with 7.5 or 10 nM of SB715992 plus 30 μM of genistein. Finally, PC-3 cells were pre-treated with 30 μM of genistein for 24 hours followed by treatment with 15 nM of SB715992. Control cells were treated with 0.3 mM Na_2_CO_3 _(vehicle control). After treatment, PC3 cells were incubated at 37°C with MTT (0.5 mg/ml, Sigma, St. Louis, MO, USA) for 2 hours and isopropyl alcohol at room temperature for 1 hour. The spectrophotometric absorbance of each sample was then determined by using ULTRA Multifunctional Micro Plate Reader (TECAN, Durham, NC, USA) at 595 nm.

### Histone/DNA ELISA for detecting apoptosis

Apoptotic cell death was quantified with the use of Cell Apoptosis ELISA Detection Kit (Roache, Palo Alto, CA, USA). PC-3 cells were seeded in 6 well plates at a density of 5.0 × 10^4^/well and allowed 24 hours to adhere to the surface of each well. Then, the cells were treated with varying concentrations of reagents as described above. After treatment, cytoplasmic histone/DNA fragments from PC-3 cells were extracted and adhered to an immobilized anti-histone antibody plate. Thereafter, a peroxidase-conjugated anti-DNA antibody was used for detection of adhered histone/DNA fragments. A substrate for peroxidase was then added to each well containing each experimental condition. The spectrophotometric absorbance of each sample was then determined by using ULTRA Multifunctional Micro Plate Reader (TECAN, Durham, NC, USA) at 405 nm.

### DNA ladder analysis for detecting apoptosis

PC-3 cells were seeded in 100 mm dishes at 3.5 × 10^5 ^cells/dish and allowed to adhere and grow for 36 hours. Following growth and attachment, PC-3 cells were treated with 15 nM of SB715992 for 48 and 72 hours. After treatment, cellular cytoplasmic DNA was extracted using 10 mM Tris (pH 8.0), 0.5 mM EDTA, and 0.2% Triton X-100. The lysate was then centrifuged at 4°C for 15 minutes at 13,800 × g to separate the cytoplasmic DNA fragments from the nuclear pellet. The supernatant was then collected and treated with 15 μl of RNase and incubated at 37°C for 1 hour. Following incubation, the supernatant was treated with 20 μl of 20% SDS, 8 μl of proteinase K (20 mg/ml), 25 μl of 5.0 M NaCl and allowed to incubate at 37°C for 30 minutes. Thereafter, phenol/chloroform/isoamyl-alcohol extraction and isopropyl alcohol precipitation were carried out. After precipitation, the DNA fragments were washed in 70% alcohol and separated through a 1.5% agarose gel at 100 volts for 80 minutes. After electrophoresis, running gels were stained with ethidium bromide and visualized by ultra-violet light.

### Microarray analysis for gene expression profiles

PC-3 cells were treated with 10 nM of SB715992 for 6, 24, and 48 hours respectively. Total RNA was extracted from each sample by the use of Trizol (Invitrogen, Carlsbad, CA) following manufacture's protocol. The total RNA of each sample was then purified with RNeasy Mini Kit and RNase-free DNase Set (Qiagen, Valenica, CA) following manufacturer's protocol. The purified RNA samples were subject to microarray anaylsis using Human Genome U133A Array (Affymetrix, Santa Clara, CA), which contains 54,613 human gene probes. Gene expression was then quantified by using Microarray Suite, MicroDB™, and Data Mining Tool Software (Affymetrix, Santa Clara, CA). Clustering and annotation of the gene expression were analyzed by using Cluster and TreeView [8], Onto-Express [9], and GenMAPP [10].

### Analysis of RNA expression by reverse transcription-polymerase chain reaction

To verify the alterations of gene expression at the mRNA level, which appeared on the microarray, we chose representative genes (Table 1 and Table 2) with varying expression profiles for real-time RT-PCR analysis. PC-3 cells were treated with 10 nM of SB715992 for 6 and 48 hours and total RNA was isolated and purified as mentioned above. Two micrograms of total RNA from each sample were subjected to reverse transcription using the Superscript first strand cDNA synthesis kit (Invitrogen, Carlsbad, CA) according to the manufacturer's protocol. Real-time PCR reactions were then carried out in a total of 25 μL reaction mixture (2 μl of cDNA, 12.5 μl of 2× SYBR Green PCR Master Mix, 1.5 μl of each 5 μM forward and reverse primers, and 7.5 μl of H_2_O) in SmartCycler II (Cepheid, Sunnyvale, CA). The PCR program was initiated by 10 min at 95°C before 40 thermal cycles, each of 15 s at 95°C and 1 min at 60°C. Data were analyzed according to the comparative Ct method and were normalized by actin expression in each sample. Melting curves for each PCR reaction were generated to ensure the purity of the amplification product.

### Western blot analysis

PC-3 cells were seeded into 100 mm culture dishes at 3.0 × 10^5 ^cells per dish and allowed to attach overnight for 24 hours. The cells were treated with 10 nM of SB715992 for 24 and 48 hours. The cells were then lysed in 62.5 mM Tris-HCl and 2% SDS. The protein concentrations of each sample were measured using a BCA Protein Assay Kit (PIERCE, Rockford IL). The cellular protein extracts were then subjected to 10 or 14% of SDS-PAGE and transferred to nitrocellulose membranes at 100 V for 2 hours at 4°C. The membranes were incubated with anti-EGFR (1:500, Santa Cruz, CA), anti-p27 (1:100, Santa Cruz, CA), and anti-p15 (1:200, Santa Cruz, CA), and anti-β-actin (1:10000, Sigma, MO) primary antibodies, and subsequently incubated with secondary antibodies conjugated with peroxidase. The signal was then detected using Chemiluminescent Detection System (PIERCE, Rockford, IL).

### Statistical analysis

For cell growth inhibition assay and apoptosis ELISA, statistical analysis was performed using *t *test between treated and untreated samples or between monotreatment and combination treatment. *P *values less than 0.05 indicate statistical significance.

## Results

### Inhibition of PC-3 prostate cancer cell proliferation by SB715992

**Figure 1:**
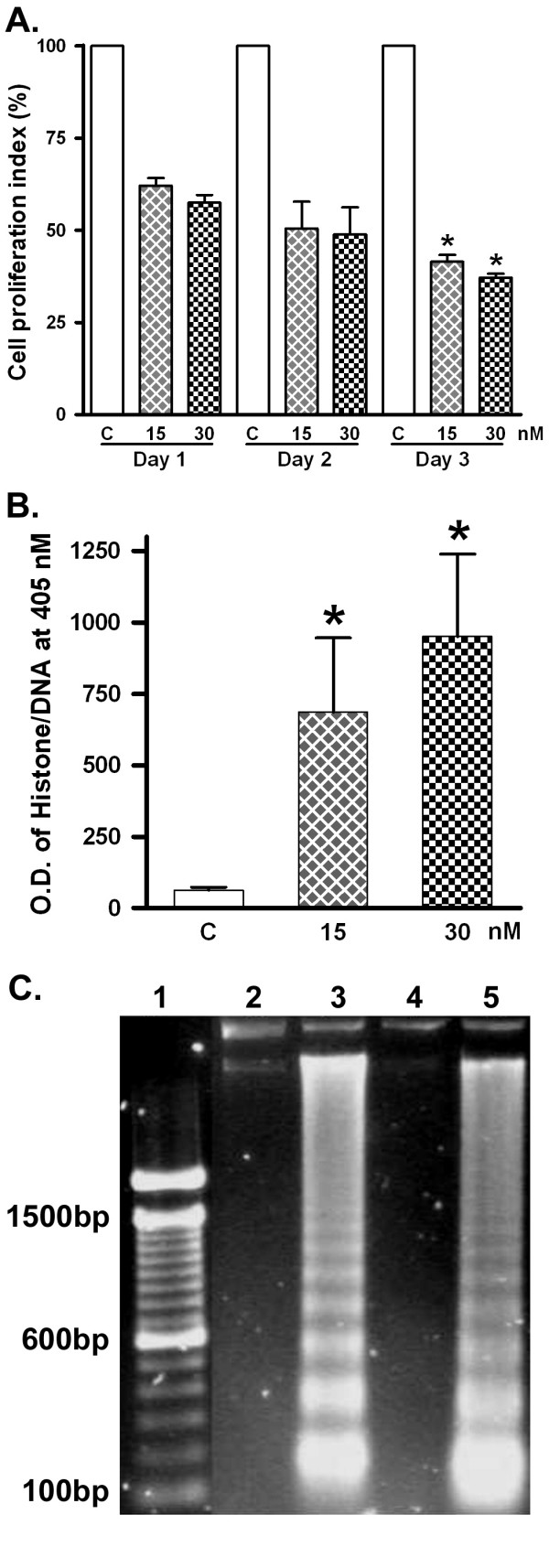
**A. **Inhibition of PC-3 cell proliferation analyzed by MTT assay. PC-3 cells were treated with 15 and 30 nM of SB715992 respectively (*:*P *< 0.001 compared with control, n = 12). **B. **Induction of apoptosis in PC-3 cells by SB715992 assayed by ELISA. PC-3 cells were treated with 15 and 30 nM of SB715992 for 24 to 72 hours (*:*P *< 0.05, n = 3). **C. **Induction of apoptosis in PC-3 cells by SB715992, measured by DNA-ladder analysis. PC-3 cells were treated with 15 nM of SB715992. (Lanes 1: 100 bp DNA marker, 2: Control 48 hours, 3: Treatment 48 hours, 4: Control 72 hours, 5: Treatment 72 hours).

PC-3 prostate cancer cells were treated with 15 and 30 nM of SB715992 and assayed for inhibition of cell proliferation. Results obtained from MTT analysis showed that SB715992 had a time and dose related effect on the growth of PC-3 cells (Figure 1a). Over the course of 72 hours, SB715992 caused an average increase in cell growth inhibition of 48.65% at 15 nM and 52.16% at 30 nM with respect to control samples. Therefore, the results from this experiment suggested that SB715992 is a potent inhibitor of PC-3 cell growth *in vitro*. Given this information, we directed our investigation on the ability of SB715992 to induce apoptosis in PC-3 prostate cancer cells.

### Induction of apoptosis in prostate cancer cell by SB715992

The induction of apoptosis was tested by two different methods. First, the induction of apoptosis by SB715992 was detected by ELISA assay. The results obtained from the ELISA assay showed an average of 1094.88% increase in apoptosis when PC-3 cells were treated with 15 nM of SB715992 and 1516.70% when treated with 30 nM of SB715992 for 72 hours (Figure 1b). As a result, SB715992 had also shown a time and dose related effect on the increase of apoptosis, which was correlated with our results obtained from MTT assay. Therefore, we decided to confirm our ELISA data by performing a DNA ladder analysis. The results from the DNA ladder analysis also showed a time related increase in apoptosis induced by SB715992 (Figure 1c). Thus, the results from both the ELISA assay and DNA ladder analysis showed clearly that SB715992 was a strong inducer of apoptosis in prostate cancer cells. As a result, the information obtained from the assays for apoptosis and cell proliferation inhibition prompted us to investigate the changes in gene expression and the regulation caused by treatment of PC-3 prostate cancer cells with SB715992.

### Regulation of RNA expression by SB715992

The gene expression profile of PC-3 cells exposed to SB715992 was accessed by microarray analysis using Human Genome U133A Array. Of the 54,613 genes, a total of 120 at 6 hours, 418 at 24 hours, and 1713 at 48 hour were up-regulated after SB715992 treatment. Our data also showed that 126 genes at 6 hours, 110 at 24 hours, and 1264 at 48 hours were down regulated. After clustering and annotation of the gene expression, we selected 34 genes with the most significant changes with respect to categories such as apoptosis, cell cycle, cell proliferation, cell signaling, and protein kinase. Our results showed an up-regulation of genes that induce apoptosis and inhibit cell cycle progression and cell signalling (Table 1). Our results also showed a down regulation of genes related to cell survival, such as protein kinase, growth factors, transcription, and translation (Table 1). To confirm the data from microarray, we conducted RT-PCR analysis on 16 of the 34 genes selected from the microarray analysis. The results from our RT-PCR analysis were in mutual agreement with the results obtained from the microarray analysis (Table 3). The data obtained from both RT-PCR and microarray analysis showed clearly that SB715992 up regulated genes that are responsible for apoptosis and cell cycle arrest, and down regulated genes that are responsible for cell proliferation and survival. As a result, alterations in RNA expression of PC-3 cell by SB715992 led us to investigate the alteration in protein expression of selected critical genes, which are expressed in prostate cancer cells and are important for cell cycle regulation and cell growth.

### Regulation of protein expression in prostate cancer cells by SB715992

**Figure 2:**
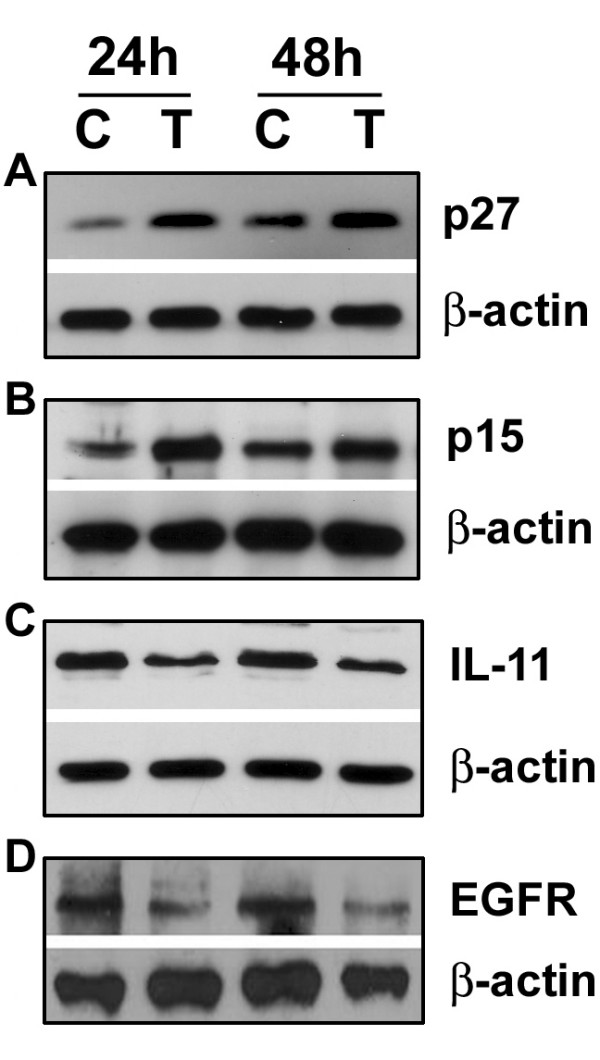
Alteration in protein expression in PC-3 cells treated with SB715992 and analyzed by Western Blot analysis. PC-3 cells were treated with 10 nM of SB715992 (**A. **p27/β-actin; **B. **p15/β-actin; **C. **IL-11/β-actin; **D. **EGFR/β-actin;).

In order to investigate the alteration in protein expression in PC-3 cells, Western Blot analysis was conducted. Our results showed a qualitative decrease in the expression of EGFR after 48 hours of SB715992 (Figure 2a), suggesting a down regulation of EGFR gene. Our results also showed a qualitative increase in the expression of p27 (CDKN2B) and p15 (CDKN1B) (Figure 2b and 2c), suggesting an up-regulation of these genes. These results were in direct agreement with the results obtained from microarray analysis and RT-PCR analysis. Therefore, our results obtained by different assays clearly suggest that SB715992 regulates the expression of genes that are vital for cell proliferation and apoptosis. Because we previously found that genistein also regulated the expression of genes that are critical for the control of cell growth and apoptosis, we investigated whether combination treatment with genistein and SB715992 could exert more inhibitory effects on PC-3 prostate cancer cell growth and induce greater degree of apoptotic cell death compared to either agent alone.

### Genistein increased anti-proliferation activity of SB715992

**Figure 3:**
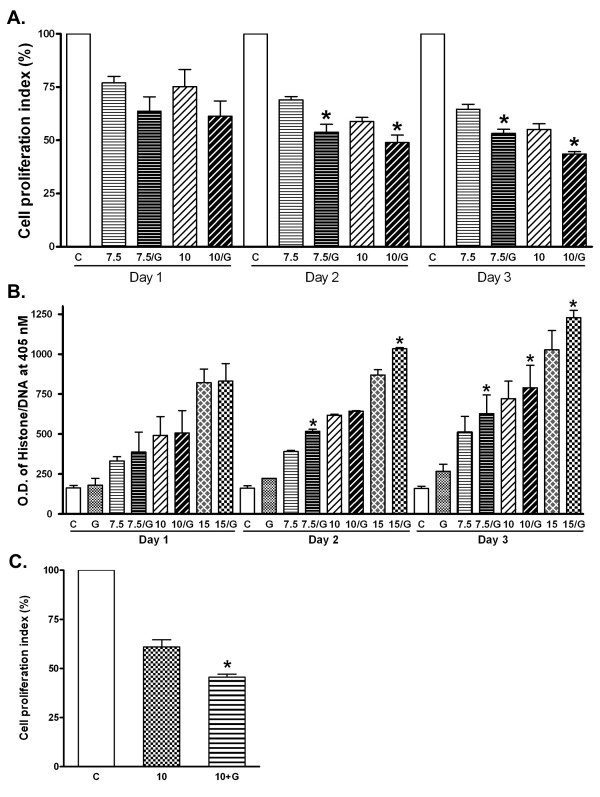
**A. **Inhibition of PC-3 cell proliferation was enhanced by genistein as measured by MTT assay. G indicates treatment with 30 μM of genistein (*: *P *< 0.05 compared with SB715992 treatment alone, n = 5). **B. **Induction of apoptosis in PC-3 cells was enhanced by genistein. G indicates treatment with 30 μM of genistein. (*: *P *< 0.05 compared with SB715992 treatment alone, n = 3.). **C. **Inhibition of PC-3 cell proliferation was enhanced by pre-treatment with genistein. G indicates pre-treatment with 30 μM of genistein. (*: *P *< 0.05 compared with SB715992 treatment alone. n = 8).

To investigate whether genistein could potentiate the growth inhibitory effects of SB715992, PC-3 cells were treated with a combination of SB715992 and genistein, and assayed for inhibition of cell proliferation by MTT analysis (Figure 3a). Our results showed an average increase in growth inhibition of 29.83% when treated with 7.5 nM of SB715992, and 36.99% when treated with 10 nM of SB715992 over 72 hours. However, when the cells were treated with 7.5 nM SB715992 plus 30 μM of genistein, we found 48.73% growth inhibition over 72 hours. Thus, combining SB715992 with genistein increased the percentage of cell growth inhibition compared to mono-treatments. These results led us to investigate whether pre-treatment with genistein could sensitize PC-3 cells to the growth inhibitory effects of SB715992. We pre-treated PC-3 cells with 30 μM of genistein for 24 hours. After the pre-treatment, we exposed the cells to 15 nM of SB715992 for 24 and 72 hours. Our results showed a greater degree of cell growth inhibition compared to cells treated simultaneously with these agents (Figure 3a). We subsequently tested whether genistein could increase the apoptotic inducing effect of SB715992.

### Genistein enhanced pro-apoptotic effect of SB715992

To test for induction of apoptosis, PC-3 cells were treated with 30 μM of genistein, 7.5 nM of SB715992 with 30 μM of genistein, and 10 nM of SB715992 with 30 μM of genistein. PC-3 cells were also treated with 10, 15, and 30 nM of SB715992 for direct comparison (Figure 3b). Our results showed that an average increase in apoptosis was 38.59% when treated with genistein compared to untreated cells over the course of 72 hours. Also, we observed a 7.44% increase in apoptosis when treated with 10 nM of SB715992 combined with 30 μM genistein with respect to cells only treated with SB715992. These ELISA results were correlated with those obtained by MTT analysis, suggesting that genistein not only induces cell growth inhibitory activity of SB715992, but also caused induction of apoptotic cell death.

## Discussion

Cancer chemotherapeutic agents, which target spindle and perturb mitosis, have been shown to be clinically effective for the treatment of cancers. These agents are clinically used mainly for deregulating one motor protein, tubulin. However, kinesin spindle protein (KSP) is another important motor protein, which makes up a larger family of microtubules and forms bipolar spindle with other motor proteins. This motor protein plays an essential role in nuclear motility and mitotic spindle functions [7,11]. The mitotic spindle, commonly referred to as the nuclear spindle, is composed of spindle fibers of which some are adhered to chromosomes positioned at centromeres. This effect is apparently involved in chromosomal movement [12,13]. The mitotic spindle is also composed of continuous fibers that stretch and pass from pole to pole forming a bipolar spindle. These bipolar spindles are required for proper cell division [14,15]. Thus, it is critical that assembly of the spindles is accurate and that they are maintained with high fidelity. Recently, researchers have used this information to produce chemical agents to enhance cessation of the cell cycle and induce apoptosis in mitotic cells, with their principle target being the *Homo sapiens Eg5 *kinesin spindle protein (*HsEg5*/KSP) [16]. If the functions of *HsEg5*/KSP can be reversed or inhibited, this could prove to be a novel way to manipulate the cell cycle and cell proliferation in mitotic cells [7]. In this study, we found that SB715992, an experimental KSP inhibitor, significantly inhibited the proliferation of PC-3 human prostate cancer cells at nanomole concentration, suggesting that this inhibitor of KSP may be a potent agent for the treatment of prostate cancer.

Current literature has shown that inhibition of KSP perturbs mitosis and leads to cell death [7,16-20]. The inhibition of KSP influences the formation of bipolar spindle that is required for proper separation and lining of the chromosomes during mitosis. This abnormality in chromosomes leads to programmed cell death in mitotic cells [21]. Here, we showed that SB715992 significantly induced apoptotic cell death in PC-3 prostate cancer cells, suggesting that SB715992 could inhibit the formation of bipolar spindle during cell mitosis, resulting in apoptotic cell death of PC-3 cells.

From gene expression profiles altered by SB715992, we found that cellular and molecular responses to SB715992 treatment are complex and are likely to be mediated by a variety of regulatory pathways. SB715992 regulated the expression of important genes that control cell growth, apoptosis, transcription, translation, and cell signaling. These regulations may be responsible for inhibiting the progression of prostate cancers. It has been well known that cyclins associate with cyclin-dependent protein kinases (CDKs) and CDK inhibitors to control the process of cell cycle. The CDK inhibitors such as p27^KIP1^, p15, and p57^Kip2 ^have been demonstrated to arrest the cell cycle and inhibit the growth of cancer cells. From gene expression profiles, we found that SB715992 increased the expression of several cyclin-dependent kinase inhibitors including p27^KIP1^, p15, and p57^Kip2^, suggesting a positive change in promotion of cyclin-dependent kinase inhibitors, which could ultimately lead to cell cycle arrest (Fig. 4) [23,24]. On the other hand, SB715992 decreased the expression of genes such as fibroblast growth factor (FGF) and epidermal growth factor (EGFR) and these genes are important molecules in favour of cell survival and proliferation. Therefore decrease in the expression of these genes could negatively regulate cell cycle progression (Fig. 4), cell proliferation, angiogenesis, motility, metastasis, and cell signaling [22].

Another objective in this study was to investigate whether genistein, a naturally occurring isoflavone, could potentiate the effects of SB715992 on human prostate cancer cells. Previously, genistein has been shown to inhibit nuclear transcription factor, NF-κB and Akt signaling pathways in cancer cells, leading to apoptosis [21,25-29]. Genistein has also been shown to inhibit angiogenesis and topoisomerase I & II. Our study showed that genistein increased the growth inhibitory effects of SB715592 on PC-3 cells. In addition, we also found that genistein could also enhance the induction of apoptosis in PC-3 cells induced by SB715992, suggesting that genistein could be clinically useful when combined with SB715992. Therefore, we believe that SB715992 could be used as a novel therapeutic agent for prostate cancer and that the pre-treatment of patients with genistein prior to administration of SB715992 could even be a better therapeutic strategy with lowering systemic toxicity caused by SB715992. However, further in-depth investigations including *in vivo *study are needed in order to establish cause and effect relationships between these altered genes and treatment outcome in animal models as well as in human patients.

## Competing interests

The author(s) declare that they have no competing interests.

## Authors' contributions

FHS designed the study and prepared the manuscript. DAD carried out cell growth inhibition, apoptosis assays, microarray and Western Blot analysis and drafted the manuscript. SHS carried out real-time PCR. MH participated in the design of the study. YL carried out Western Blot analysis and modified the manuscript. All authors read and approved the manuscript.

## Pre-publication history

The pre-publication history for this paper can be accessed here:



## Figures and Tables

**Table 1:** Fold changes in selected genes treated with SB715992 tested by microarray analysis.

			**Fold Change**		
			
**Accession Number**	**Gene Symbol**	**Description**	**6 h**	**24 h**	**48 h**
R64130	PPBP	pro-platelet basic protein (chemokine (C-X-C motif) ligand 7)	1.07	1.74	6.50
AI050866	NODAL	nodal homolog (mouse)	1.41	5.28	9.19
NM_001106	ACVR2B	activin A receptor, type IIB	5.66	6.50	11.31
NM_005118	TNFSF15	tumor necrosis factor (ligand) superfamily, member 15	1.23	4.92	11.31
NM_005228	EGFR	epidermal growth factor receptor	-1.52	-1.15	-2.00
BC001971	CDKN1B	cyclin-dependent kinase inhibitor 1B (p27, Kip1)	1.32	1.23	3.03
BC000125	TGFB1	transforming growth factor, beta 1 (Camurati-Engelmann disease)	-1.41	-1.23	-2.00
AA994334	BCL10	B-cell CLL/lymphoma 10	1.74	2.46	12.13
AV654063	KRAS2	v-Ki-ras2 Kirsten rat sarcoma 2 viral oncogene homolog	18.38	13.00	19.70
NM_004464	FGF5	fibroblast growth factor 5	-1.32	-7.46	-6.06
AK027071	TSC22	transforming growth factor beta 1 induced transcript 4	NC	2.83	5.28
BG326045	BHLHB2	basic helix-loop-helix domain containing, class B, 2	-1.15	2.64	9.19
U73844	ELF3	E74-like factor 3 (ets domain transcription factor, epithelial-specific)	-1.07	2.64	6.96
S75264	WT1	Wilms tumor 1	-1.15	-1.41	-4.59
AW514564	CHD2	chromodomain helicase DNA binding protein 2	-1.07	3.48	14.93
AF180474	CNOT3	CCR4-NOT transcription complex, subunit 3	-1.52	-2.14	-4.29
N95363	CDKN1C	cyclin-dependent kinase inhibitor 1C (p57, Kip2)	-2.00	1.23	6.06
AL039795	TARDBP	TAR DNA binding protein	1.52	4.29	10.56
AK022513	DUSP10	dual specificity phosphatase 10	-1.32	3.73	8.57
AA994004	CDK11	cyclin-dependent kinase (CDC2-like) 11	8.57	8.00	128.00
L78790	ITGB2	integrin, beta 2 (antigen CD18 (p95)	-1.23	-1.32	-10.56
NM_003954	MAP3K14	mitogen-activated protein kinase kinase kinase 14	-1.15	-1.41	-2.64
NM_030662	MAP2K2	mitogen-activated protein kinase kinase 2	-1.32	-1.32	-2.00
AW025150	MAP3K12	mitogen-activated protein kinase kinase kinase 12	1.32	-1.15	-2.00
AW450910	SYK	spleen tyrosine kinase	-1.52	-1.52	-14.93
M57765	IL11	interleukin 11	1.07	-2.00	-4.59
AW444761	CDKN2B	cyclin-dependent kinase inhibitor 2B (p15, inhibits CDK4)	1.07	6.06	9.19
NM_002031	FRK	fyn-related kinase	4.29	6.96	25.99

**Table 2:** List of primer sequences used for RT-PCR analysis.

**Genes**	**Forward Primer Sequence**	**Reverse Primer Sequence**	**PCR Product Size**
IL11	agctgagggacaaattcc	cacacctgggagctgtag	104 b.p.
CDK11	caaacggaaaactggatg	caggtgtccttgaatgct	148 b.p.
FRK	ccagctccatttgatttg	ttatctgtgcctccctca	202 b.p
p15	cacaatggagctagaagca	aattccattttcgaagcc	153 b.p.
CHD2	aagggactccaaggaatg	aggtttgcatttgtatgctt	192 b.p.
CNOT3	tggaacgagagaccaaaa	aatccggtcctgcttatc	218 b.p.
p57	gaccgttcatgtagcagc	caccttgggaccagtgta	142 b.p.
ELF3	gagtcggaactgagggtt	tgaggaggcaccagataa	286 b.p.
ACVR2B	tcgaagtagagctgtggc	catgcaggtatgagaggc	138 b.p.
TNFSF15	ccacctattttgtgctgg	agatgatccacccacctt	211 b.p.
EGFR	gtgctggatgatagacgc	attgttgctggttgcact	286 b.p.
p27	tggtgatctcccaagcta	aaaactcccaagcacctc	189 b.p.
KRAS2	cagggcgaatttgtaatg	gttcagtagggcagctca	229 b.p.
FGF	ttgtccttcctcctcctc	agggttcctatcagtggc	117 b.p.

**Table 3:** Comparative gene expression levels from microarray and RT-PCR analysis

**Gene**	**Description**	**Microarray 48 h Fold Change**	**RT-PCR 48 h Fold Change**
IL-11	interleukin 11	-4.59	-2.13
CDK11	cyclin-dependent kinase (CDC2-like) 11	128.00	4.03
FRK	fyn-related kinase	25.99	11.00
p15	cyclin-dependent kinase inhibitor 2B (p15, inhibits CDK4)	9.19	5.41
CHD2	chromodomain helicase DNA binding protein 2	14.93	15.74
CNOT	CCR4-NOT transcription complex, subunit 3	-4.29	-5.01
p57	cyclin-dependent kinase inhibitor 1C (p57, Kip2)	6.06	35.84
ELF3	E74-like factor 3 (ets domain transcription factor)	6.96	24.53
ACVR	activin A receptor, type IIB	11.31	2.95
TNFSF	tumor necrosis factor (ligand) superfamily, member 15	11.31	30.00
EGFR	epidermal growth factor receptor	2.00	6.00
p27	cyclin-dependent kinase inhibitor 1B (p27, Kip1)	3.03	10.90
KRAS2	v-Ki-ras2 Kirsten rat sarcoma 2 viral oncogene homolog	19.7	3.56
FGF	fibroblast growth factor	-6.06	-5.26
